# Naproxen-Derived New Compound Inhibits the NF-κB, MAPK and PI3K/Akt Signaling Pathways Synergistically with Resveratrol in RAW264.7 Cells

**DOI:** 10.3390/molecules28083395

**Published:** 2023-04-12

**Authors:** Yi Ou, Zonglin You, Min Yao, Yingfan Cao, Xiu Xue, Min Chen, Rihui Wu, Lishe Gan, Dongli Li, Panpan Wu, Xuetao Xu, Wingleung Wong, Vincent Kam Wai Wong, Wenfeng Liu, Jiming Ye, Jingwei Jin

**Affiliations:** 1 School of Biotechnology and Health Sciences, Wuyi University, Jiangmen 529020, China; 2 International Healthcare Innovation Institute (Jiangmen), Jiangmen 529040, China; 3 Neher’s Biophysics Laboratory for Innovative Drug Discovery, State Key Laboratory of Quality Research in Chinese Medicine, Macau University of Science and Technology, Macau 999078, China; 4 Lipid Biology and Metabolic Disease Research Group, School of Health and Biomedical Sciences, RMIT University, Melbourne, VIC 3000, Australia

**Keywords:** naproxen derivative, resveratrol, synergistic, anti-inflammatory, mechanism

## Abstract

Naproxen is widely used for anti-inflammatory treatment but it can lead to serious side effects. To improve the anti-inflammatory activity and safety, a novel naproxen derivative containing cinnamic acid (NDC) was synthesized and used in combination with resveratrol. The results showed that the combination of NDC and resveratrol at different ratios have a synergistic anti-inflammatory efficacy in RAW264.7 macrophage cells. It was indicated that the combination of NDC and resveratrol at a ratio of 2:1 significantly inhibited the expression of carbon monoxide (NO), tumor necrosis factor α (TNF-α), interleukin 6 (IL-6), induced nitric oxide synthase (iNOS), cyclooxygenase 2 (COX-2) and reactive oxygen species (ROS) without detectable side effects on cell viability. Further studies revealed that these anti-inflammatory effects were mediated by the activation of nuclear factor kappa-B (NF-κB), mitogen-activated protein kinase (MAPK) and phosphoinositide-3 kinase (PI3K)/protein kinase B (Akt) signaling pathways, respectively. Taken together, these results highlighted the synergistic NDC and resveratrol anti-inflammatory activity that could be further explored as a strategy for the treatment of inflammatory disease with an improved safety profile.

## 1. Introduction

Inflammation produced by the self-defense system in humans and other living creatures, as an immune response, is well connected with inflammatory diseases, including metabolic and neurodegenerative diseases, as well as and peptic ulcers [1,2]. Furthermore, uncontrolled inflammation (chronic inflammation) often damages tissues and this is associated with the increased risk of cancer, such as colon, gastric and lung cancer [3]. The RAW264.7 cell line presents a typical phenotype of macrophage cells involved in processing inflammatory antigens [4,5]. Once macrophages are activated by lipopolysaccharides (LPS), a large number of inflammatory mediators and cytokines, including carbon monoxide (NO), tumor necrosis factor α (TNF-α), interleukin 6 (IL-6) and reactive oxygen species (ROS), are induced to synthesize and release in macrophages [6,7,8]. It is confirmed that the cytokines from macrophages are believed to be the key contributors to inflammatory diseases [9,10]. Currently, non-steroidal anti-inflammatory drugs (NSAIDs), including aspirin, ibuprofen and naproxen, are widely used to treat inflammatory diseases. However, long term use of such drugs (in particular naproxen) can cause a series of side effects, such as hypertension, heart failure, acute myocardial infarction, kidney injury and gastrointestinal complications [11,12,13,14,15]. Thus, it is important to develop new, safe and effective anti-inflammatory drugs.

**Figure 1:**
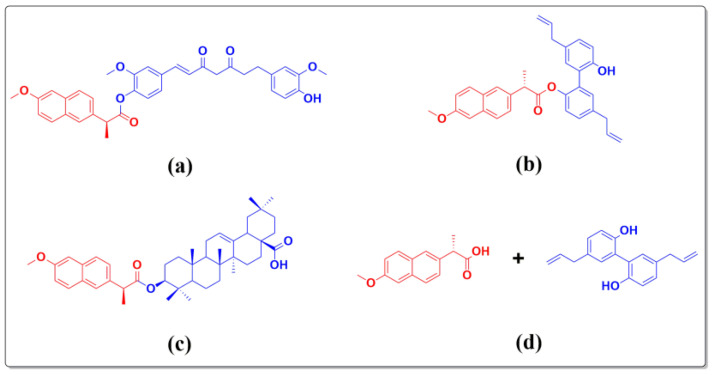
The reported naproxen derivatives and naproxen combined with magnolol. (**a**) naproxen derivative containing curcumin, (**b**) naproxen derivative containing magnolol, (**c**) naproxen derivative containing oleanolic acid, and (**d**) naproxen combined with magnolol.

Naproxen is one type of NSAID used for the treatment of inflammatory diseases, such as inflammatory bowel and rheumatic diseases, as well as myocarditis [16]. Current studies have revealed that naproxen combined with natural compounds, or structurally modified naproxen with active compounds derived from natural products are beneficial to enhance their bioactivity [17,18,19,20]. As shown in Figure 1a,b, it has been suggested that a naproxen derivative containing curcumin or magnolol reduced phorbol-12-myristate-13-acetate (TPA)-induced skin inflammation in a TPA-induced mouse ear edema model. The mechanistic studies revealed that these compounds can inhibit the over-expression of pro-inflammatory cytokines by blocking the NF-κB signaling pathway. In addition, naproxen derivatives containing oleanolic acid have a wide range of bioactivities (Figure 1c). As described in Figure 1d, the combination of naproxen and magnolol can effectively reduce TPA-induced skin inflammation [20]. Therefore, the combination of naproxen and natural compounds, or structurally modified naproxen with natural compounds are effective strategies to treat inflammatory diseases.

Based on the biological diversification of resveratrol and cinnamic acid that is isolated from its natural product [21,22], this study aims to investigate the anti-inflammatory activity and mechanisms of NDC combined with resveratrol that contains cinnamic acid, and to explore the potential therapy strategy. Firstly, NDC was synthesized, and then the effect of NDC combined with resveratrol at different concentrations was determined by using LPS-induced RAW264.7 cells. Finally, the mechanism of NDC combined with resveratrol was studied.

## 2. Results and Discussion

### 2.1. NDC Design

**Scheme 1:**
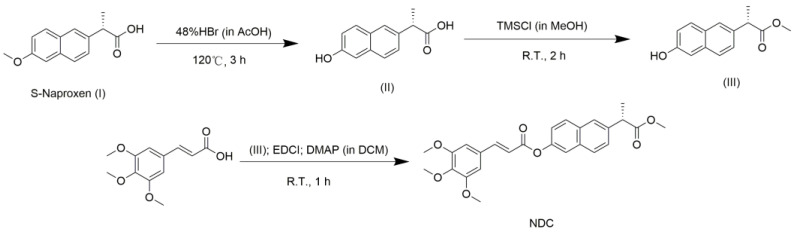
The synthetic route of NDC.

As described in a previous publication, compound III was synthesized in two steps using naproxen as the starting material [23]. As shown in Scheme 1, the NDC compound was synthesized through the esterification of compound III and 3,4,5-trimethoxycinnamic acid. As exhibited in Supplementary Figures S2–S4, The results have confirmed that NDC compound contained naproxen and cinnamic acid. The anti-inflammatory activities and mechanisms of NDC and resveratrol, as well as the combination of resveratrol and NDC, were investigated in order to explore the novel bioactive compound with a high potency.

### 2.2. Effect of NDC and Resveratrol on the RAW264.7 Cell Viability

**Figure 2:**
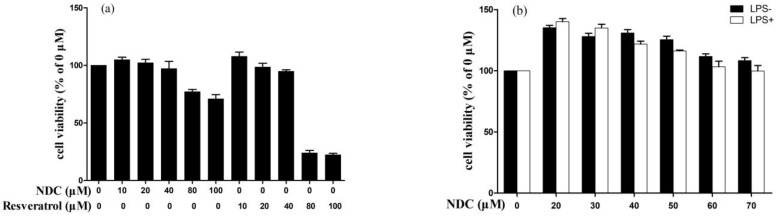

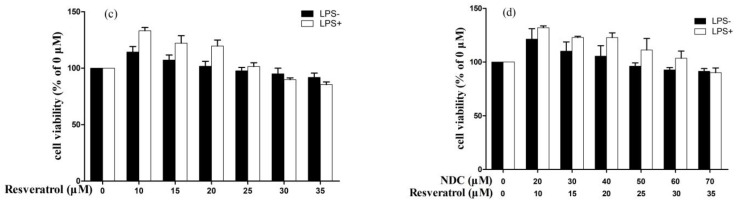
Effect of NDC and resveratrol on the viability of the RAW264.7 cells, (**a**) NDC or resveratrol (0–100 μM) without LPS, (**b**) NDC (0–70 μM) with or without LPS (2 μg/mL), (**c**) resveratrol (0–35 μM) with or without LPS (2 μg/mL), and (**d**) NDC (0–70 μM) and resveratrol (0–35 μM) in combination with or without LPS (2 μg/mL), *n* = 3.

Prior to investigating the anti-inflammatory effect of NDC and resveratrol on LPS-induced RAW264.7 cells, the cytotoxic effects of different concentrations of NDC and resveratrol were tested using a 3-(4,5-dimethythiazol-2-yl)-2,5-diphenyl-tetra-zoliumbromide (MTT) assay model. It was indicated that treatment with NDC (at a concentration of 100 μM) and resveratrol (at a concentration of 80 μM and 100 μM) without LPS (2 μg/mL), individually had an obvious cytotoxicity on the RAW264.7 cells (Figure 2a). Hence, the concentrations of NDC and resveratrol were down to 40 μM and 80 μM, respectively, for further investigation. As exhibited in Figure 2b,c, treatment with NDC (20–70 μM) and resveratrol (10–35 μM) did not produce any obvious cytotoxicity on the RAW264.7 cells with or without LPS stimulation. Moreover, treatment with the combination of NDC and resveratrol did not significantly decrease the cell viability of the RAW264.7 cells with or without LPS stimulation (Figure 2d). These results demonstrated that NDC and resveratrol, individually or in combination, at the concentrations described above did not decrease the cell viability of the RAW264.7 cells with or without LPS stimulation.

### 2.3. Effects of NDC and Resveratrol on LPS-Induced NO Production

**Figure 3:**
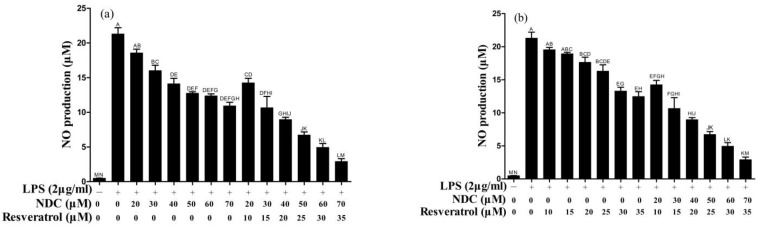
Effect of NDC on LPS-induced NO in the absence and presence of resveratrol. (**a**) NDC and a combination of NDC and resveratrol, (**b**) resveratrol and a combination of NDC and resveratrol. Groups that do not share the same letter are significantly (*p* < 0.01) different from each other. The letters are in order of average size, *n* = 3.

It was indicated that once the RAW264.7 cells were stimulated by LPS, iNOS would be released in the cells, and then a large amount of NO, that was mediated by iNOS, would become synthesized in the cells, and cause severe inflammation [24]. Thus, blocking the NO synthesis may be considered an effective strategy to improve the inflammatory reaction. In the present study, the inhibition of NDC and resveratrol on the NO levels was investigated using LPS-induced RAW264.7 cells as a model. As described in Figures S1 and Figure 3, the NO levels of the RAW264.7 cells stimulated with LPS markedly increased. In addition, NDC and resveratrol, individually or combined, dependently suppressed LPS-induced NO production (Figure 3a,b). Interestingly, NDC combined with resveratrol provided a stronger inhibition of LPS-induced NO production than NDC or resveratrol alone (Figure 3a,b). The results demonstrated that pretreatment with NDC (70 μM) and resveratrol (35 μM) individually, caused a 48.73% and 41.47% decrease in the NO levels, respectively. However, pretreatment with a combination of NDC and resveratrol resulted in an 86.31% reduction in LPS-induced NO production.

### 2.4. Synergistic Effect Analysis

**Figure 4:**
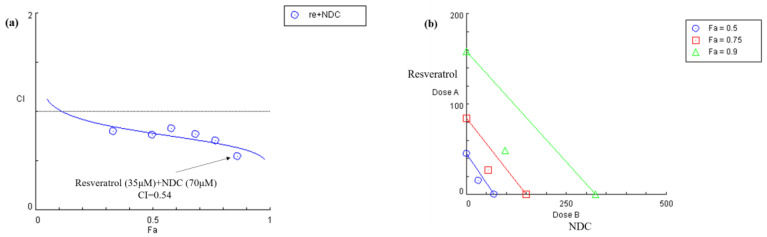
Combination index analysis for the inhibition of LPS-induced NO treated with NDC or resveratrol alone, or a combination of both. (**a**) Combination index plot, (**b**) isobologram for the combination of NDC and resveratrol.

In order to investigate whether NDC combined with resveratrol has a synergistic inhibition effect on LPS-induced NO production, the data described above were analyzed using CompuSyn software 2.0. As shown in Figure 4a, the combination index (CI) values of NDC combined with resveratrol (molar ratio of 2:1) ranged from 0.54 to 0.82. Furthermore, the results indicated that NDC (70 μM) combined with resveratrol (35 μM) had the lowest CI value (0.54). As exhibited in Figure 3b, all of NDC combined with resveratrol achieved a 90% inhibition [fraction affected (Fa) == 0.9; green triangle], 75% inhibition (Fa == 0.75; red square) and 50% inhibition (Fa == 0.5; blue circle) were below the respective lines. Taken together, it was suggested that the combination of NDC and resveratrol had the strongest synergistic inhibition effect on LPS-induced RAW264.7 cells.

Based on the results described above, NDC (70 μM) and resveratrol (35 μM) were selected for the mechanistic studies. The inhibition of NDC (70 μM) and resveratrol (35 μM), individually or in combination, against LPS-induced TNF-α, IL-6, ROS, iNOS, COX-2 and NF-κB, MAPK and PI3K/Akt signaling pathways were further determined to interpret the synergistic effect and the anti-inflammatory mechanism of NDC combined with resveratrol.

### 2.5. Effects of NDC and Resveratrol on LPS-Induced IL-6 and TNF-α

**Figure 5:**
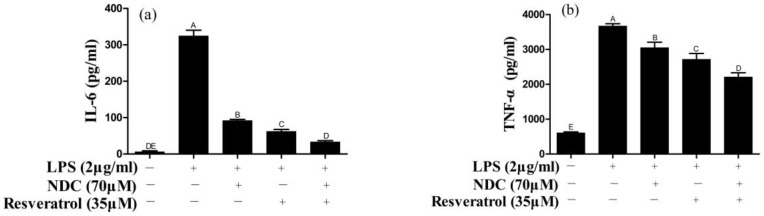
Effect of NDC and resveratrol on LPS-induced IL-6 (**a**) and TNF-α (**b**). Groups that do not share the same letter are significantly (*p* < 0.01) different from each other. The letters are in order of average size, *n* = 3.

It is well known that pro-inflammatory cytokines (IL-1β, IL-6 and TNF-α) induced by LPS play an important role in the inflammatory process and in inflammatory diseases [7]. Thus, the effect of NDC and resveratrol on IL-6 and TNF-α in LPS-induced RAW264.7 cells was determined using an enzyme-linked immunosorbent assay (ELISA). As shown in Figure 5a,b, the individual treatments with NDC and resveratrol could inhibit the LPS-induced over-expression of IL-6 and TNF-α with a value of 71.82%, 16.89%, 80.95% and 26.05%, respectively. However, treatment with NDC (70 μM) combined with resveratrol (35 μM) (the molar ratio 2:1) had the best inhibitory effect on the expression of IL-6 and TNF-α, and resulted in an 89.96% inhibition of IL-6 and a 39.77% inhibition of TNF-α (*p* < 0.01).

### 2.6. Effects of NDC and Resveratrol on LPS-Induced ROS

**Figure 6:**
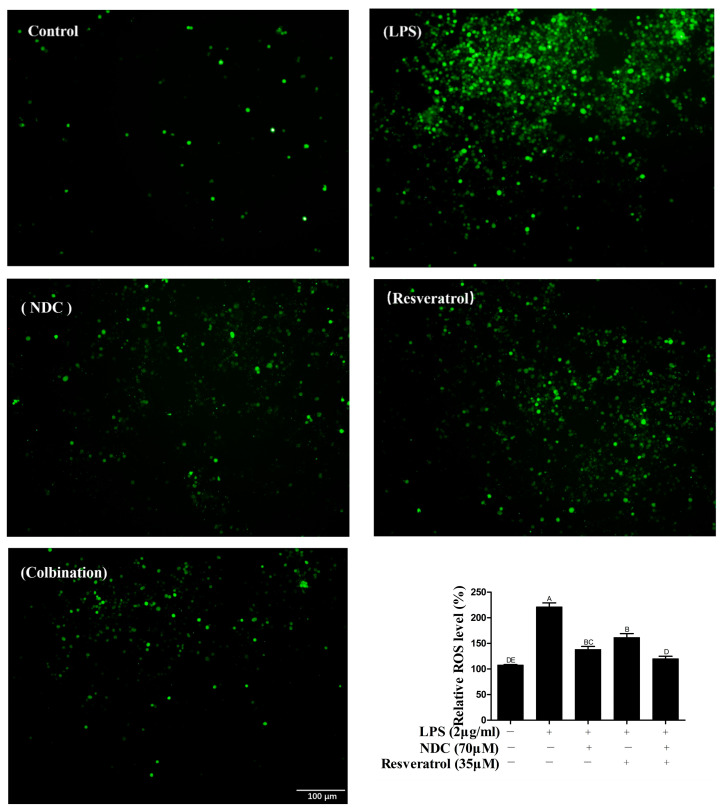
The effect of NDC and resveratrol on LPS-induced ROS in RAW264.7 cells. Side bar = 100 µm. Groups that do not share the same letter are significantly (*p* < 0.01) different from each other. The letters are in order of average size, *n* = 3.

Once stimulated by LPS, various ROS are released in inflammatory cells to activate the inflammatory signaling pathway and cause cellular and tissue damage [25]. Thus, the effect of NDC and resveratrol on LPS-induced ROS has been determined. As shown in Figure 6, the fluorescence intensity of ROS on the LPS group is remarkably enhanced in comparison to that in the control group. However, after pre-treatment with NDC and resveratrol, individually or in combination (70 μM and 35 μM respectively), the fluorescence intensity is significantly decreased in LPS-induced RAW264.7 cells. Furthermore, NDC in combination with resveratrol (70 μM and 35 μM, respectively) showed stronger effects (45.70%) than the individual treatment with NDC (37.57%) and resveratrol (26.97%) on LPS-induced ROS.

### 2.7. Effects of NDC and Resveratrol on iNOS and COX-2 Protein Expressions

**Figure 7:**
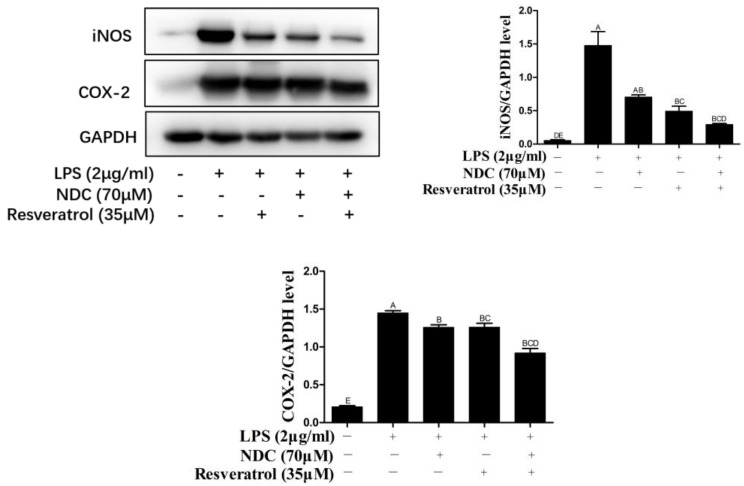
Effect of NDC and resveratrol on the expression of iNOS and COX-2 in LPS-induced RAW264.7 cells. Groups that do not share the same letter are significantly (*p* < 0.01) different from each other. The letters are in order of average size, *n* = 3.

It is confirmed that NO synthesized by iNOS, and prostaglandin E2 (PGE_2_) synthesized by COX-2, are important inflammatory mediators in inflammatory responses [26,27]. Therefore, various stimuli, including LPS and ROS, induce the over-expression of iNOS and COX-2. In order to investigate the effect of NDC and resveratrol on LPS-induced iNOS and COX-2 in RAW264.7 cells, the protein expressions of iNOS and COX-2 were tested using Western blotting. As described in Figure 7, the protein expression levels of iNOS and COX-2 in LPS-induced RAW264.7 cells were remarkably higher than those in the control group. When pretreated with NDC or resveratrol individually, the expression levels of iNOS and COX-2 had a moderate decrease in LPS-induced RAW264.7 cells (66.55% and 13%, 52.18% and 13.10%, respectively). Moreover, pretreatment with NDC (70 μM) and resveratrol (35 μM) in combination, respectively, caused an 80.06% and 36.42% decrease in iNOS and COX-2 expressions.

### 2.8. Effects of NDC and Resveratrol on the Activation of Side NF-κB, MAPK and PI3K/Akt Pathway Proteins in LPS-Induced RAW264.7 Cells

**Figure 8:**
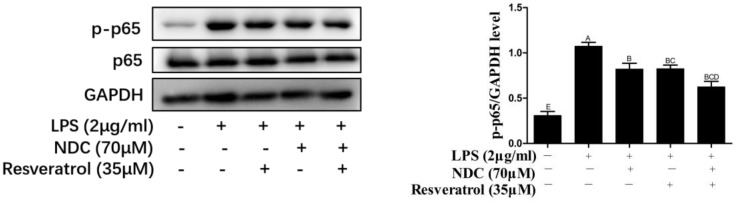
Effect of NDC and resveratrol on LPS-induced NF-κB pathways in RAW264.7 cells. Groups that do not share the same letter are significantly (*p* < 0.01) different from each other. The letters are in order of average size, *n* = 3.

It was indicated that once the RAW264.7 cells were induced by LPS, the NF-κB signaling pathway was subsequently activated and caused the phosphorylation of p65 [28]. Furthermore, the activation of the NF-κB signaling pathway could lead to the over-expression of iNOS and COX-2 in LPS-induced RAW264.7 cells [29]. Therefore, the inhibition of the NF-κB signaling pathway was a potent strategy for the treatment of inflammation and inflammatory diseases. To assess whether NDC and resveratrol could modulate the NF-κB activation, the protein expression of p65 and phosphorylated p65 (p-p65, the activated form) were determined by Western blotting. As shown in Figure 8, the protein expression level of phosphorylated p65 was significantly increased in LPS-induced RAW264.7 cells. Pretreatment with NDC (70 μM) and resveratrol (35 μM), individually or in combination, effectively decreased the LPS-induced p65 activation and phosphorylation. Furthermore, the inhibition of the p-p65 protein expression in LPS-induced RAW264.7 cells by NDC (70 μM) combined with resveratrol (35 μM) was up to 41.79%, which was significantly stronger than that caused by the individual treatment with NDC and resveratrol (23.54% and 23.71%, respectively).

**Figure 9:**
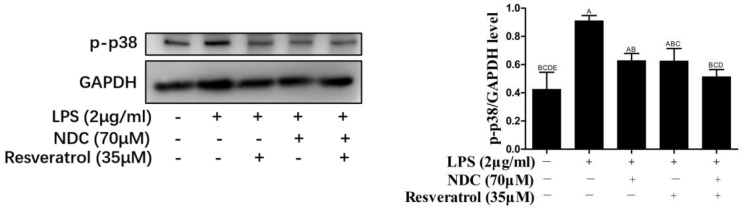
Effect of NDC and resveratrol on LPS-induced MAPK (p-p38) pathways in RAW264.7 cells. Groups that do not share the same letter are significantly (*p* < 0.01) different from each other. The letters are in order of average size, *n* = 3.

**Figure 10:**
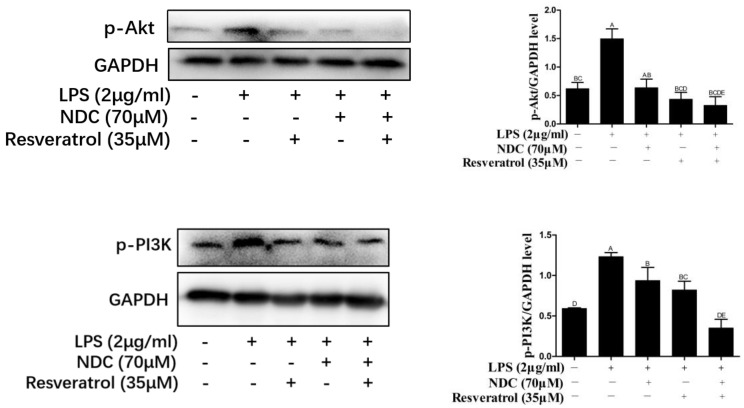
Effect of NDC and resveratrol on LPS-induced PI3K/Akt pathways in RAW264.7 cells. Groups that do not share the same letter are significantly (*p* < 0.01) different from each other. The letters are in order of average size, *n* = 3.

It was indicated that LPS could cause the phosphorylation of p38 and Akt by activating MAPK and PI3K/Akt signaling pathways, which was associated with NF-κB activity [30,31]. Hence, the levels of phosphorylated (p)-p38, p-Akt and p-PI3K were determined to evaluate the effect of NDC and resveratrol on MAPK and PI3K/Akt pathways. As shown in Figure 9 and Figure 10, the expressions of p-p38, p-Akt and p-PI3K were up-regulated in LPS-induced RAW 264.7 cells. However, pretreatment with NDC (70 μM) and resveratrol (35 μM), individually or in combination, significantly inhibited the expression of p-p38, p-Akt and p-PI3K in LPS-induced RAW 264.7 cells. NDC (70 μM) combined with resveratrol (35 μM) had the strongest inhibition in the LPS-induced expression of p-p38, p-Akt and p-PI3K.

**Figure 11:**
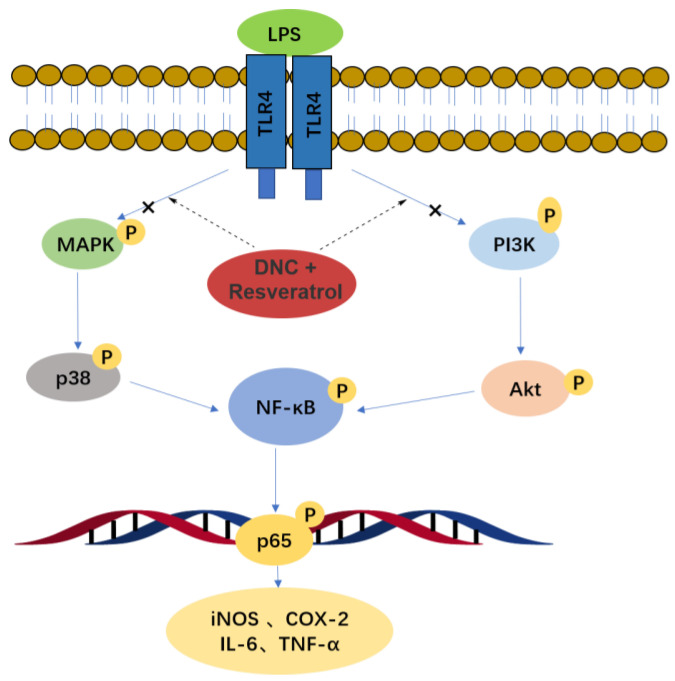
Anti-inflammatory mechanism of NDC combined with resveratrol.

It is well known that naproxen is widely used as a NSIAD for the treatment of inflammatory diseases, and it exerts anti-inflammatory activity by suppressing the over-expression of COX-2 [16]. Interestingly, the present study revealed that resveratrol and naproxen derivative had a synergistic anti-inflammatory effect in vitro. NDC (70 μM) and resveratrol (35 μM) could effectively inhibit the expression of inflammatory mediators, including NO, IL-6, TNF-α, iNOS and COX-2, and the production of ROS by suppressing NF-κB activity and blocking MAPK and PI3K/Akt signaling pathways (Figure 11).

## 3. Materials and Methods

### 3.1. Materials

NDC was synthesized and characterized in our laboratory; resveratrol and 3, 4, 5-trimethoxycinnamic acid, were purchased from Aladdin (Shanghai, China). EDCI, DMAP, S-(+)-naproxen, chlorotrimethylsilane (TMSCl) and 48% HBr solution were purchased from Macklin (Shanghai, China). CH_2_Cl_2_ and CH_3_CH_2_OH were purchased from TitanChem (Shanghai, China), Na_2_SO_4_ and CDCl_3_ were purchased from J&K Scientific (Beijing, China). Fetal bovine serum (FBS) and Dulbecco’s modified eagle medium (DMEM) were obtained from Gibco (Grand island, NY, USA). LPS (Escherichia coli O111:B4) and MTT dye were supplied by Sigma-Aldrich (St. Louis, MO, USA). Griess reagent kit was purchased from Biotium (Fremont, CA, USA). ROS assay kit was purchased from Thermo Fisher Scientific (Waltham, MA, USA). PAGE gel preparation kit was purchased from Dingguochangsheng (Beijing, China). Antibodies against COX-2, iNOS, NF-κB p65, MAPK p38, ERK, PI3K, Akt, p-p65, p-p38, p-ERK, p-PI3K and p-Akt were obtained from Affinity Biosciences (Cincinnati, OH, USA), respectively. RAW264.7 cell (p5–p9) line was obtained from Procell (Wuhan, China). ^1^H nuclear magnetic resonance (NMR) and ^13^C NMR spectra were recorded using tetramethylsilane (TMS) as an internal standard on a Bruker DPX-500 spectrometer. Mass spectrometry analysis was performed on a liquid chromatography–mass spectrometer (LC-MS, LCQ^TM^) with an ESI source. The melting points were determined on a micro melting point apparatus (Shanghai, China) and were not corrected.

### 3.2. Synthesis of NDC

As described in a previous publication, compound III was synthesized in two steps [23]. The synthesis procedure of the NDC compound was shown as follows: 3,4,5-trimethoxycinnamic acid (238 mg, 1 mmol), compound III (230 mg, 1 mmol), 1-ethyl-3-(3-dimethylaminopropyl)-carbodiimide (EDCI, 230 mg, 1.2 mmol)) and 4-dimethylaminopyridine (DMAP, 12.2 mg, 0.1 mmol) were dissolved in 30 mL of CH_2_Cl_2,_ and the reaction was magnetically stirred at room temperature for 1 h. The synthesis procedure of NDC was monitored by thin-layer chromatography. Once the reaction was completed, the solution was extracted by CH_2_Cl_2_, then it was washed twice with saturated NaHCO_3_ solution, and then dried over anhydrous Na_2_SO_4_. Following the removal of the solvent, the crude product was obtained and recrystallized in absolute ethanol to offer the NDC compound. NDC compound, white solid, yield: 53.3%, mp: 91.7 °C. ^1^H NMR (500 MHz, CDCl_3_) δ 7.90–7.84 (m, 2H, 2×Nap-H), 7.82 (d, *J* = 8.5 Hz, 1H, Nap-H), 7.78 (d, *J* = 1.7 Hz, 1H, Nap-H), 7.64 (d, *J* = 2.3 Hz, 1H, Nap-H), 7.49 (dd, *J* = 8.5, 1.8 Hz, 1H, -CH=C), 7.33 (dd, *J* = 8.9, 2.4 Hz, 1H, Nap-H), 6.86 (s, 2H, 2×Ph-H), 6.62 (dd, *J* = 15.9, 0.7 Hz, 1H, =CH-C=O), 3.94 (dd, *J* = 4.3, 0.7 Hz, 9H, 3×Ph-O-CH_3_), 3.91 (d, *J* = 7.1 Hz, 1H,-CH), 3.70 (d, *J* = 0.7 Hz, 3H,-C-O-CH_3_), 1.62 (d, *J* = 7.2 Hz, 3H, -CH_3_). ^13^C NMR (125 MHz, CDCl_3_) δ 174.93 (-C=O), 165.56 (=CH-C=O), 153.53 (-Ph-C-O), 148.48 (Nap-C-O), 146.72 (-CH=CH), 140.49 (Nap-CH), 137.94 (Nap-CH), 132.94 (Nap-C-C), 131.46 (Nap-C-C), 129.65 (Nap-C-C), 129.33 (Nap-CH), 128.15 (Nap-CH), 126.51 (Nap-CH), 126.08 (Ph-C-CH), 121.56 (Nap-CH), 118.40 (=CH-C=O), 116.44 (Nap-CH), 105.46 (Ph-CH), 61.05 (Ph-O-CH_3_), 56.21 (Ph-O-CH_3_), 52.16 (-O-CH_3_), 45.56 (-CH), 18.56 (-CH_3_). HRMS (ESI) m/z: calcd for C_26_H_26_O_7_, 451.1796 [M+ H]^+^; found, 451.1748.

### 3.3. Cell Culture

RAW264.7 cells were cultured in DMEM medium supplemented with 10% heat-inactivated FBS and antibiotics (penicillin and streptomycin). Cells (p5–p9) were maintained at sub-confluence in a CO_2_ incubator at 37 °C.

### 3.4. Cell Viability Assay

The cytotoxicity of NDC and resveratrol was tested on RAW264.7 cells using an MTT assay. Briefly, RAW264.7 cells in logarithmic growth stage were firstly seeded in 96-well plates at a density of 5 × 10^3^ cells/well. Following 24 h of culture, they were treated with NDC (10 μM, 20 μM, 40 μM, 80 μM and 100 μM) and resveratrol (10 μM, 20 μM, 40 μM, 80 μM and 100 μM) alone or in combination in a dose range of NDC (0–70 μM) and resveratrol (0–35 μM) for 2 h. Secondly, they were cultured for 24 h with or without LPS (2 μg/mL), and the cells were directly added to each well 10 μL MTT (5 mg/mL), and continued to incubate for 4 h. Finally, the supernatant was discarded after culture, and 100 μL dimethylsulphoxide (DMSO) was added to each well to dissolve the formazan crystal formed by the MTT reduction in the pore plate, and the absorbance was measured at 570 nm with a microplate reader. The control group was treated with DMSO, and all experiments were performed at least three times. Results are presented as a percentage of the controls.

### 3.5. Measurement of NO in RAW264.7 Cells

The NO level was measured using a Griess assay. Briefly, RAW264.7 cells at the logarithmic growth stage were seeded in 96-well plates at a density of 5 × 10^4^ wells. Following 24 h of culture, RAW264.7 cells were, respectively, incubated with NDC (10 μM, 20 μM, 30 μM, 40 μM, 50 μM, 60 μM and 70 μM) and resveratrol (10 μM, 15 μM, 20 μM, 25 μM, 30 μM and 35 μM), alone or their combination, in a dose range of NDC (10–70 μM) and resveratrol (10–35 μM) for 2 h. All wells were treated with LPS (2 μg/mL) except for the blank group. Twenty-four hours later, the supernatant was taken and the NO content was measured according to the instructions of the Griess reagent kit.

### 3.6. Synergistic Effect Analysis

In order to evaluate the potential synergistic anti-inflammatory effects of NDC and resveratrol at different concentrations with no toxic effects on RAW264.7 cells, the inhibitory effects of NDC (10 μM, 20 μM, 30 μM, 40 μM, 50 μM, 60 μM and 70 μM) and resveratrol (10 μM, 15 μM, 20 μM, 25 μM, 30 μM and 35 μM) on NO production in LPS-induced RAW264.7 cells were measured in the present study. The isobologram curve CI values and the synergistic inhibitory effects of NDC (10–70 μM) combined with resveratrol (10–35 μM) on the NO levels were calculated and analyzed with Compusyn 2.0 software. In addition, the strength and nature of drug-drug interaction could be quantitatively judged using the value of CI (CI > 1 as antagonism, CI = 1 as additive, 0.7 < CI < 1 as slight synergy, 0.3 < CI < 0.7 as synergy, and CI < 0.3 as strong synergy) [32].

### 3.7. ELISA Assay

RAW264.7 cells at the logarithmic growth phase were seeded at a density of 5 × 10^5^ cells per well in 96-well plates. Following 24 h of culture, RAW264.7 cells were, respectively, incubated with NDC (10 μM, 20 μM, 30 μM, 40 μM, 50 μM, 60 μM and 70 μM) and resveratrol (10 μM, 15 μM, 20 μM, 25 μM, 30 μM and 35 μM), alone or in combination, in a dose range of NDC (0–70 μM) and resveratrol (0–35 μM) for 2 h. All wells were treated with LPS (2 μg/mL) except for the blank group. The content of IL-6 in the supernatant was measured according to the instructions of the ELISA kit (Neobioscience, Shenzhen, China) after 6 h, and the content of TNF-α in the supernatant was determined after 24 h.

### 3.8. Measurement of ROS

RAW264.7 cells at the logarithmic growth phase were seeded in 96-well plates at a density of 5 × 10^5^ cells/well. Following 24 h of culture, RAW264.7 cells were, respectively, incubated with NDC (70 μM) or resveratrol (35 μM) alone, or NDC (70 μM) combined with resveratrol (35 μM) for 2 h. All wells were treated with LPS (2 μg/mL) except for the blank group. Following 24 h of induction, 2′,7′-dichlorodihydrofluorescein diacetate (H_2_DCFH-DA) was diluted with serum-free DMEM to a final concentration of 10 μM, incubated at 37 °C for 30 min without light, then washed six times with PBS, and finally the fluorescence intensity was determined at an excitation wavelength of 488 nm and emission wavelength of 525 nm with Bio-RAD.

### 3.9. Protein Extraction and Western Blot Analysis

RAW264.7 cells were seeded in a 6-well plate at a density of 1 × 10^6^ cells per well. Twenty-four hours later, the RAW264.7 cells were pretreated with NDC (70 μM) or resveratrol (35 μM) alone, or NDC (70 μM) combined with resveratrol (35 μM) for 2 h, and LPS at a dose of 2 μg/mL was added to induce an inflammatory response. The total protein was extracted by lysis of cells with a cell lysis reagent at 1 h (PI3K, Akt), 6 h (p-p38, p-p65) and 12 h (iNOS, COX-2), respectively. The protein content in lysate was determined using a BCA protein analysis kit. The total protein was separated by sodium dodecyl sulfate polyacrylamide gel electrophoresis (SDS-PAGE) and transferred to the polyvinylidene fluoride membrane. Then, the sample was blocked with 5% (*w*/*v*) skim milk powder at 4 °C overnight, and then shaken with specific primary antibodies for 2 h at room temperature, followed by incubation with horseradish peroxidase (HRP) tagged secondary antibodies for 1 h at room temperature. Finally, the imprinting was detected using enhanced chemiluminescence (ECL) and automatic radiography.

### 3.10. Data and Statistical Analysis

All analyses were performed using the GraphPad software (San Diego, CA, USA). These data were presented as the mean ± SEM except where indicated, and were generated from at least three independent experiments. The statistical analysis was conducted by using the two-way ANOVA method followed by a Tukey test using Graphpad Prism. Samples with *p* < 0.05 between the compound groups and LPS groups were considered statistically significant.

## 4. Conclusions

In summary, the present study demonstrated that NDC and resveratrol (70 μM and 35 μM molar ratio, 2:1, respectively) could effectively inhibit the over-expression of NO, IL-6 and TNF-α, as well as the release of ROS, iNOS and COX-2. It was found that the synergistic anti-inflammatory effect of NDC combined with resveratrol was associated with the inhibition of NF-κB activity and the activation of MAPK and PI3K/Akt signaling pathways. It was concluded that the combination of natural constituents and structural modifications of drugs would be a potential strategy for the treatment of inflammatory diseases.

## Data Availability

Data are also reported in the Supplementary Materials.

## Supplementary Materials

The following supporting information can be downloaded at: https://www.mdpi.com/article/10.3390/molecules28083395/s1, Figure S1: The inhibition of different compounds at 50 μ M on NO production. Figure S2: 1 H NMR spectra of NDC. Figure S3: 1 3 C NMR spectra of NDC. Figure S4: HR MS spectra of NDC.

## Sample Availability Statement

Samples of the compounds are available from the authors.
