# Supplementary material for: Naproxen-Derived New Compound Inhibits the NF-κB, MAPK and PI3K/Akt Signaling Pathways Synergistically with Resveratrol in RAW264.7 Cells
Source: Molecules. 2023 Apr 12;28(8):3395. doi: 10.3390/molecules28083395 (PMC10146875; doi:10.3390/molecules28083395)
Supplement: Supplementary file 1 [file molecules-28-03395-s001.zip › molecules-2242671-supplementary.pdf]

Supplementary data

Naproxen-derived new compound inhibits the NF-κB, MAPK and PI3K/Akt signaling pathway synergistically with resveratrol in RAW264.7 cells

Yi Ou [1, a], Zonglin You [1, a], Min Yao[a], Yingfan Cao [a], Xiu Xue [a], Min Chen [a], Rihui Wu[a, b], Lishe Gan [a, b], Dongli Li [a, b], Panpan Wu[a, b], Xuetao Xu [a, b], Wing-Leung Wong [a, b], Vincent Kam Wai Wong [c], Wenfeng Liu[*, a, b], Ji-Ming Ye[*, a, d], Jingwei Jin[*, a, b]

[a] *School of Biotechnology and Health Sciences, Wuyi University, Jiangmen 529020, China.*
[b] *International Healthcare Innovation Institute (Jiangmen), Jiangmen 529040, P. R. China.*
[c] *Dr. Neher's Biophysics Laboratory for Innovative Drug Discovery, State Key Laboratory of Quality Research in Chinese Medicine, Macau University of Science and Technology, Macau, China.*
[d] *Lipid Biology and Metabolic Disease Research Group, School of Health and Biomedical Sciences, RMIT University, Melbourne, Australia.*

* Corresponding author:
Wenfeng Liu, Telephone: +86 13189852897, E-mail: wyuchemlwf@126.com
Ji-Ming Ye, Telephone: +61423965894, E-mail: jiming.ye@kbimed.com
Jingwei Jin, Telephone: +86 15017523457, E-mail: wyuchemjjw@126.com
[1] Yi Ou and Zonglin You contributed equally to this work.

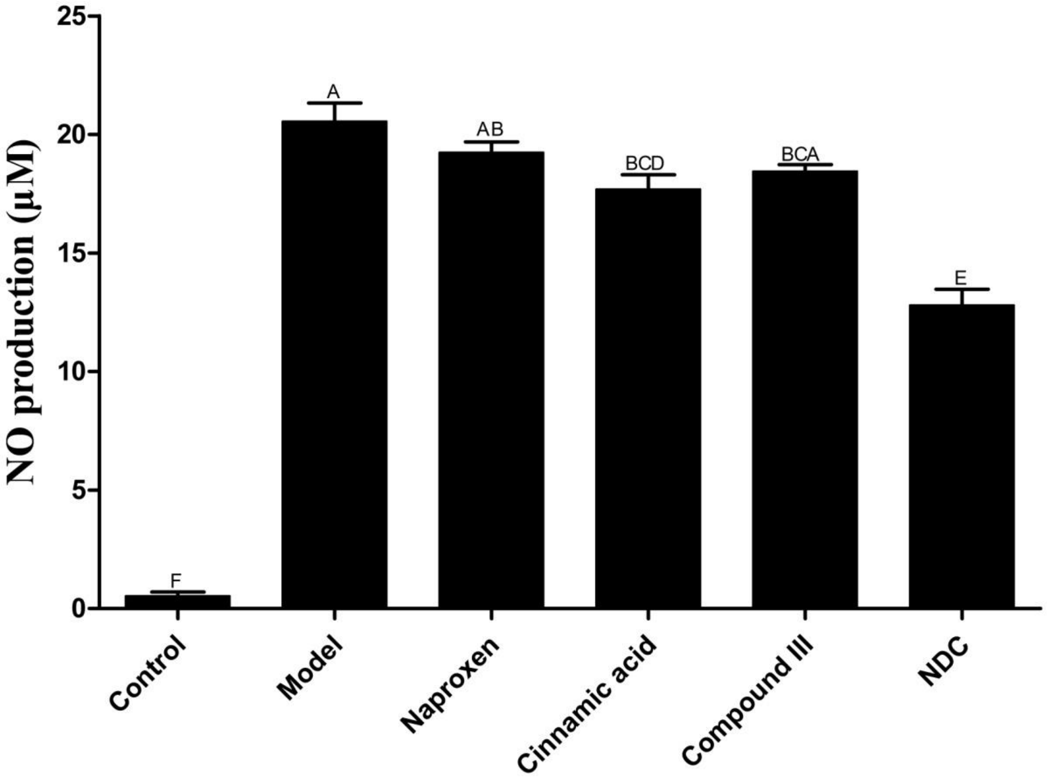

Figure S1. The inhibition of different compounds at 50 μM on NO production.

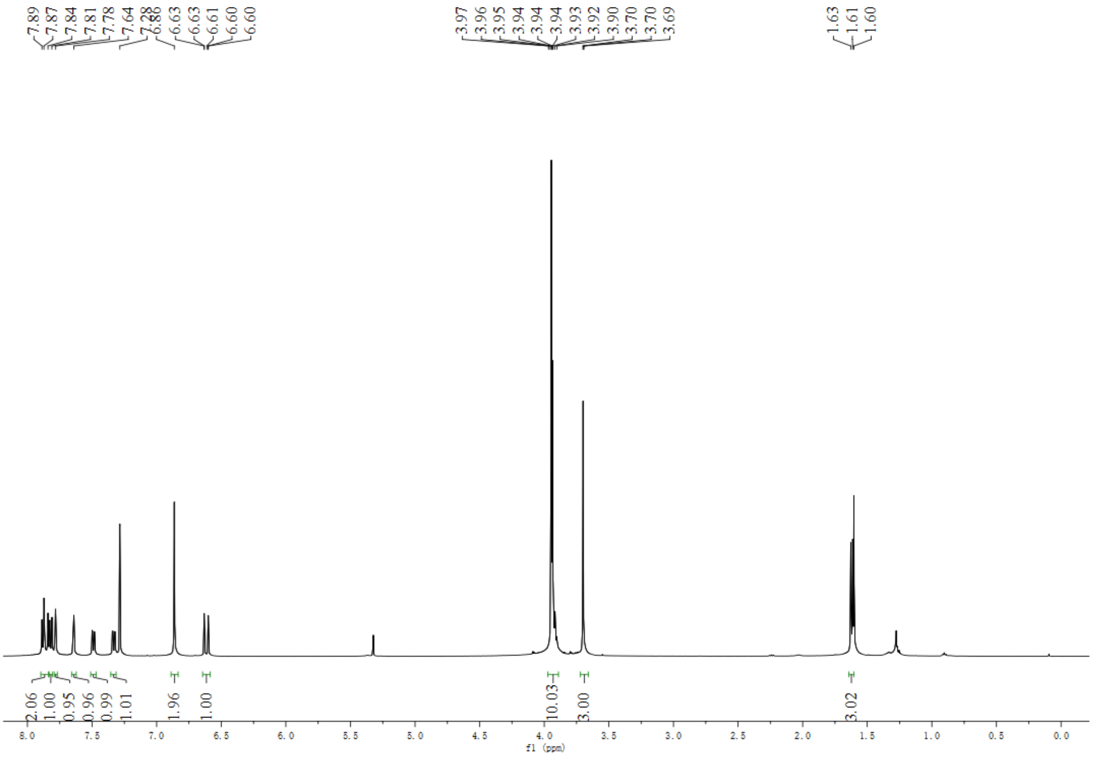

Figure S2. ^{1}H-NMR spectra of NDC

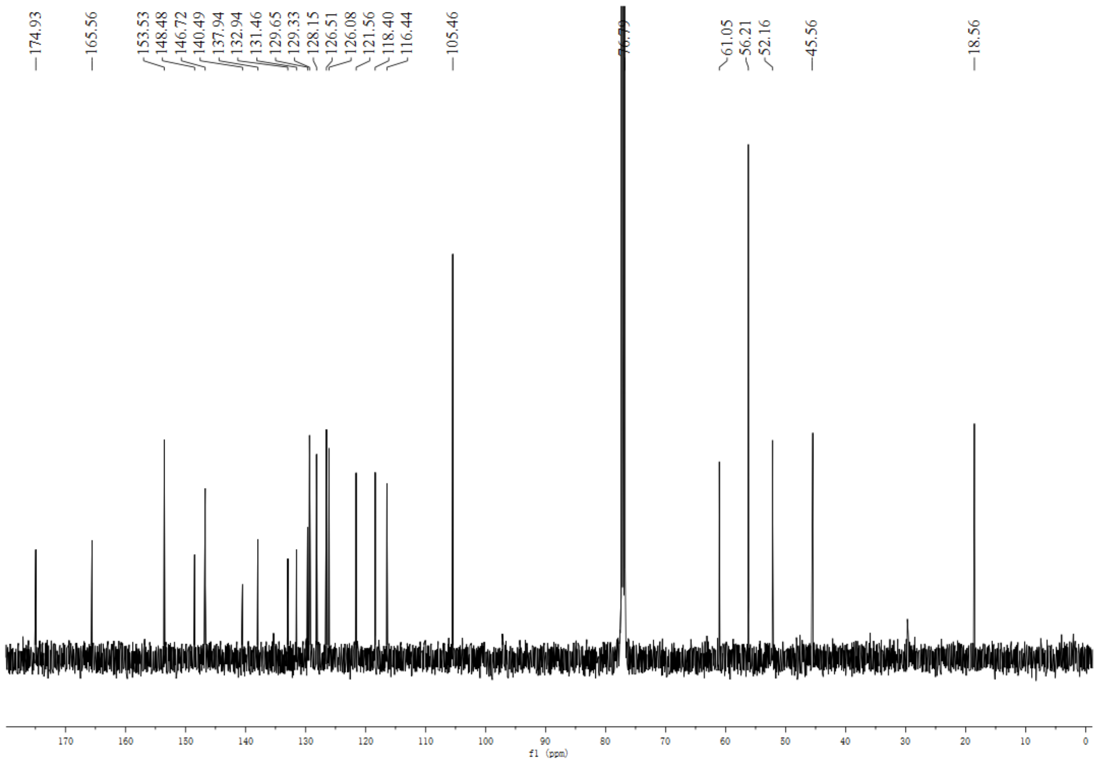

Figure S3. ^{13}C-NMR spectra of NDC

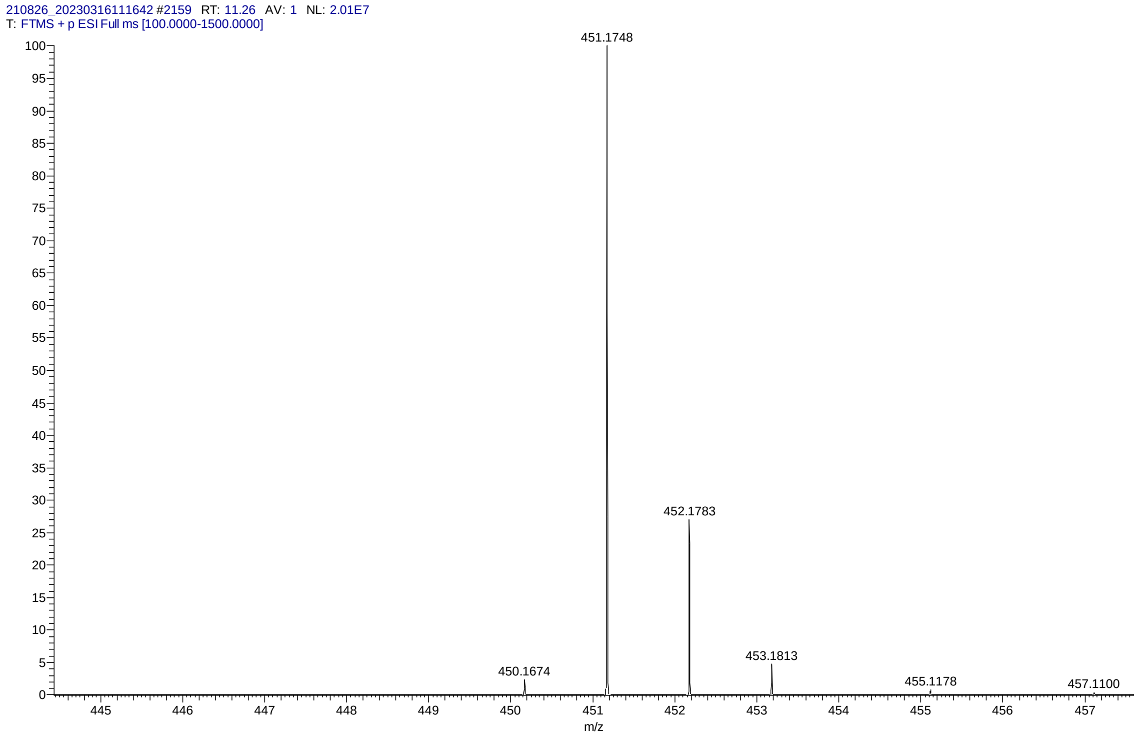

Figure S4. HR-MS spectra of NDC
